# Explosive multi-site metastasis temporally associated with acute exacerbation of idiopathic pulmonary fibrosis in a patient with surgically resected lung squamous cell carcinoma: a case report

**DOI:** 10.3389/fmed.2026.1874765

**Published:** 2026-07-07

**Authors:** Junyi Mu, Yi Ding, Yunxia Yu, Rong Jiang, Zhijun Jie, Jingjing Feng

**Affiliations:** 1 Department of Pulmonary and Critical Care Medicine, Shanghai Fifth People's Hospital, Fudan University, Shanghai, China; 2 Center of Community-Based Health Research, Fudan University, Shanghai, China

**Keywords:** acute exacerbation, hypoxia, idiopathic pulmonary fibrosis, lung squamous cell carcinoma, tumor metastasis

## Abstract

We report a rare case of a 78-year-old male with lung squamous cell carcinoma (SCC) and concurrent idiopathic pulmonary fibrosis (IPF) who underwent thoracoscopic partial left upper lobectomy and systematic mediastinal lymph node dissection. The tumor remained stable under regular surveillance for 13 months postoperatively. An acute exacerbation of IPF (AE-IPF) then occurred, resulting in severe hypoxemia. Although the patient’s condition temporarily stabilized following antifibrotic therapy and high-flow nasal cannula (HFNC) oxygen therapy, explosive multi-organ metastases appeared within one year of AE-IPF onset, successively involving the liver, abdominal wall, and brain. The unique features of this case are: (1) a clinically “silent” interval of more than one year following curative surgery, after which severe systemic hypoxia accompanying AE-IPF was temporally associated with subsequent explosive tumor dissemination and is hypothesized to be a potential predisposing factor for rapid tumor spread; and (2) rare metastatic patterns for lung SCC, including in abdominal wall subcutaneous metastasis and suspected acral (digital) metastasis whose distinction from a primary trichilemmal carcinoma could not be definitively resolved. This case underscores the importance of early antifibrotic therapy to prevent AE-IPF, maintaining adequate oxygenation, and implementing comprehensive multidisciplinary surveillance for patients with lung cancer comorbid with IPF.

## Introduction

Idiopathic pulmonary fibrosis (IPF) is a chronic, progressive, fibrosing interstitial lung disease with a poor prognosis and a median survival of only 3–5 years (1). Epidemiological and mechanistic studies have confirmed that IPF is an independent risk factor for lung cancer, with both diseases sharing core pathogenic mechanisms including chronic epithelial injury, aberrant tissue repair, epithelial-mesenchymal transition (EMT), and a pro-oncogenic fibrotic microenvironment. The cumulative incidence of lung cancer in patients with IPF reaches 3.3, 15.4, and 54.7% at 1, 5, and 10 years from IPF diagnosis, respectively. This incidence rate is substantially higher than in the general population (2, 3). Among all lung cancer subtypes in IPF, squamous cell carcinoma is the most common histological type, accounting for approximately 37.8% of cases (3). Patients with concurrent lung cancer and IPF constitute a particularly challenging clinical population owing to severely impaired pulmonary reserve, limited therapeutic options, and the constant risk of AE-IPF (4, 5).

AE-IPF is defined by an acute, clinically significant deterioration in respiratory function with bilateral radiological worsening superimposed on the usual interstitial pneumonia (UIP) pattern, in the absence of an identifiable alternative cause (1, 6). The median survival following AE-IPF is only 3–4 months, with in-hospital mortality as high as 50% (1). Beyond the direct respiratory threat, severe systemic hypoxia induced by AE-IPF may promote tumor progression through upregulation of hypoxia-inducible factor-1α (HIF-1α), induction of EMT, stimulation of angiogenesis, and suppression of anti-tumor immunity, thereby potentially accelerating tumor invasion and distant metastasis (7, 8). However, published case reports documenting explosive multi-site tumor dissemination likely linked to AE-IPF-induced systemic hypoxia remain exceedingly scarce.

We present a case of a 78-year-old male with surgically resected early-stage lung SCC and co-existing IPF who, following AE-IPF-induced severe hypoxemia, rapidly developed multi-organ metastases at sites rare for lung SCC, including the liver, brain, abdominal wall, and digits, and subsequently died of hemorrhagic cerebral metastasis. The temporal association between AE-IPF and explosive tumor dissemination, together with the uncommon metastatic distribution, raises important questions regarding the potential role of systemic hypoxia in metastatic dissemination in this unique patient population, and highlights the critical need for early antifibrotic intervention and multidisciplinary management.

## Case presentation

### Initial diagnosis and surgical treatment

A 78-year-old man with a 40-pack-year smoking history (ceased 10 years prior to presentation) presented in August 2023 with a one-week history of cough and blood-tinged sputum. High-resolution computed tomography (HRCT) of the chest revealed two solid nodules in the left upper lobe (maximum diameters approximately 2.5 cm and 1.8 cm), highly suspicious for malignancy (Figure 1A). Simultaneously, HRCT demonstrated bilateral reticular opacities, traction bronchiectasis, and basal honeycombing consistent with a usual interstitial pneumonia (UIP) pattern. The patient had no history of occupational dust exposure, connective tissue disease, or drug-induced lung injury. Based on integrated clinical and radiological assessment according to current diagnostic guidelines, a diagnosis of IPF was established (1).

**Figure 1:**
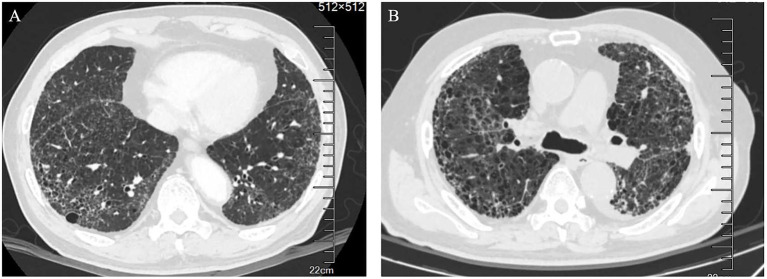
Chest HRCT images. **(A)** Preoperative CT (August 2023) demonstrating two solid nodules in the left upper lobe (arrows), bilateral reticular opacities, traction bronchiectasis, and basal honeycombing consistent with a UIP pattern, accompanied by emphysema. **(B)** CT at the time of AE-IPF (September 2024) showing marked bilateral progression of fibrosis with extensive new ground-glass opacities superimposed on the pre-existing UIP pattern and worsening honeycombing.

CT-guided percutaneous transthoracic needle biopsy of the left upper lobe nodule confirmed moderately differentiated squamous cell carcinoma. Preoperative pulmonary function testing revealed: FEV1 1.85 L (65% of predicted), DLCO 58% of predicted. Cranial CT and abdominal ultrasonography revealed no obvious metastatic lesions, after multidisciplinary team (MDT) evaluation, the patient was deemed to have adequate physiological reserve for surgical resection. He subsequently underwent video-assisted thoracoscopic surgery (VATS) partial left upper lobectomy combined with systematic mediastinal lymph node dissection. Final pathological staging was pT2aN0M0 (Stage IIA, AJCC 8th edition), with negative surgical margins and 0/15 lymph nodes involved. Pathological examination confirmed a moderately differentiated squamous cell carcinoma arising from the apical segment of the left upper lobe, measuring 4.5 × 3.5 × 1.5 cm (maximum diameter: 4.5 cm). No visceral pleural invasion, lymphovascular invasion, or spread through air spaces was identified. Immunohistochemically, the tumor was positive for CK, CK7, P40, and CK5/6, and negative for TTF-1 and Napsin A, with a Ki-67 proliferation index of 60%. Given the patient’s impaired baseline pulmonary function and underlying IPF, adjuvant chemotherapy was deferred following MDT discussion, given the risk of triggering chemotherapy-induced AE-IPF (4, 5). Regular postoperative surveillance was scheduled every three months. Tumor markers remained within normal limits and chest CT demonstrated no evidence of recurrence or metastasis throughout the first 13 months of follow-up. Notably, despite the established diagnosis of IPF at the time of surgery, antifibrotic therapy was not initiated postoperatively.

### Acute exacerbation of IPF

In September 2024 (13 months postoperatively), the patient developed fever (38.5 °C), progressive dyspnea, and severe hypoxia without an obvious identifiable etiology. Physical examination revealed resting oxygen saturation (SpO_2_) of approximately 80% on room air, and bilateral basal Velcro crackles on auscultation. Arterial blood gas analysis (nasal cannula oxygen at 5 L/min): PaO_2_ 67 mmHg, PaCO_2_ 35 mmHg, and PaO_2_/FiO_2_ ratio approximately 160 mmHg. Emergency chest HRCT demonstrated marked bilateral progression of pulmonary fibrosis with extensive new ground-glass opacities superimposed on the pre-existing UIP pattern, accompanied by worsening honeycombing (Figure 1B). Laboratory investigations revealed markedly elevated serum Krebs von den Lungen-6 (KL-6) at 5,782 U/mL (reference<1,000 U/mL), lactate dehydrogenase (LDH) 345 U/L, C-reactive protein (CRP) 56 mg/L, and procalcitonin (PCT) 0.08 ng/mL. The results of B-type natriuretic peptide (BNP),sputum culture and D-dimer were all negative. Bases on progressive dyspnea, and severe hypoxia, and potential alternative etiologies including infectious pneumonia, heart failure, and pulmonary embolism were systematically excluded. A diagnosis of AE-IPF was established in accordance with current consensus criteria (1, 6).

The patient received intravenous methylprednisolone pulse therapy (500 mg/day×3 days, subsequently tapered to oral prednisolone 30 mg/day) combined with high-flow nasal cannula (HFNC) oxygen therapy (FiO_2_ 60%, flow rate 40 L/min). Dyspnea and hypoxemia gradually improved, with SpO_2_ stabilizing at 92–94% on nasal cannula oxygen at 3 L/min. Upon discharge, the patient was prescribed long-term oxygen therapy (LTOT) and commenced nintedanib 150 mg twice daily as antifibrotic treatment (9, 10).

### Explosive multi-site metastasis and terminal course

Within one year of AE-IPF onset, the patient successively developed the following multi-site lesions, representing an explosive pattern of disease dissemination:

1) Abdominal wall and hepatic metastases (August 2025):The patient reported dull upper abdominal discomfort. Physical examination revealed a firm, fixed, non-tender subcutaneous nodule measuring approximately 3*2 cm in the upper abdominal wall. Contrast-enhanced abdominal CT demonstrated multiple hypodense hepatic lesions of varying sizes (largest approximately 4.2 cm), consistent with metastases, and a subcutaneous soft-tissue nodule in the upper abdominal wall. Abdominal ultrasonography confirmed a hypoechoic subcutaneous nodule measuring 27 × 17 mm with rich internal vascularity. Ultrasound-guided core needle biopsy of the abdominal wall nodule revealed metastatic poorly differentiated carcinoma; immunohistochemistry demonstrated CK5/6(+), P40(+), and P63(+), confirming a squamous cell origin consistent with metastatic lung SCC (Figures 2A,B). Serum squamous cell carcinoma antigen (SCC-Ag) was elevated at 6.8 ng/mL (reference < 1.5 ng/mL), while carcinoembryonic antigen (CEA) was normal.
2) Digital lesions (April–June 2025): In April 2025, firm, erythematous, markedly tender nodules with localized swelling appeared sequentially on the distal phalanx of the right little finger and the left thumb (Figures 3A,B). Plain radiographs demonstrated osteolytic destruction of the distal phalanges of both affected digits (Figures 3C,D). Amputation of the distal phalanx of the left thumb was performed in June 2025; histopathological examination revealed a malignant neoplasm with morphological features favoring trichilemmal carcinoma (Figures 2C,D). However, given the concurrent systemic tumor progression evidenced by hepatic and abdominal wall SCC metastases, the osteolytic radiographic features atypical of a primary cutaneous adnexal tumor, and the established association between lung SCC and acral metastasis (11, 12), the possibility of digital acrometastasis from the primary lung SCC could not be definitively excluded.
3) Pulmonary recurrence (August–November 2025): Sequential chest CT demonstrated a newly emerged soft-tissue mass in the left thoracotomy bed that progressively enlarged over a three-month interval (Figures 4A–C), accompanied by further worsening of bilateral pulmonary fibrosis.
  Given the patient’s severely impaired performance status (ECOG PS 4), profound oxygen dependency (SpO_2_ 90–92% on nasal cannula oxygen at 5 L/min at rest), and inability to tolerate any systemic antitumor therapy—platinum-doublet chemotherapy was associated with a 5–12% risk of fatal AE-IPF (4, 5); immune checkpoint inhibitors carried a substantially elevated risk of ICI-related pneumonitis in the setting of pre-existing IPF (13); and actionable oncogenic drivers are rarely identified in lung SCC [14]-the MDT determined that the goals of care should shift to best supportive treatment (continued oxygen therapy, nintedanib, antimicrobial therapy as needed, nutritional support, and analgesics).
4) Cerebral metastases with hemorrhage (November 2025): The patient experienced a sudden decline in consciousness and a hypertensive crisis (systolic blood pressure 220 mmHg). Emergency non-contrast CT of the head demonstrated a large left fronto-parieto-temporal hemorrhage (estimated volume ≈ 60 mL) with significant midline shift (≈ 12 mm), and scattered hyperdense nodular lesions within the brain parenchyma consistent with hemorrhagic metastases (Figure 4D). The patient’s condition continued to deteriorate, and he died of transtentorial herniation secondary to cerebral hemorrhage in December 2025, approximately five months after the initial detection of systemic metastases and 15 months after the AE-IPF episode (Table 1).

**Figure 2:**
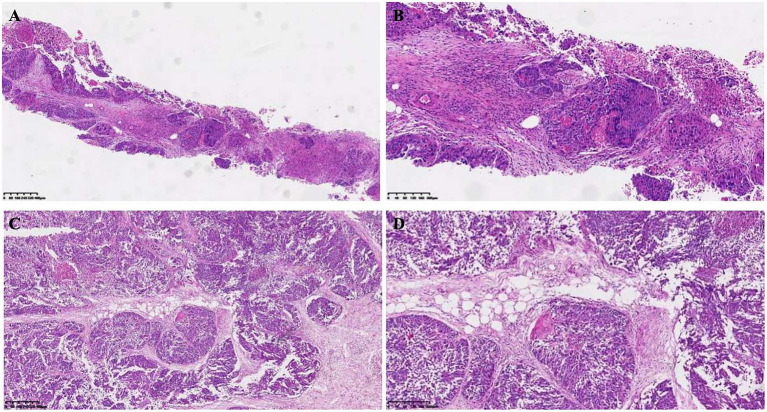
Histopathological images. **(A)** Abdominal wall core needle biopsy (H&E, ×4); **(B)** Abdominal wall core needle biopsy (H&E, ×10) demonstrating nests of atypical squamous cells with readily identifiable mitotic figures, consistent with metastatic poorly differentiated squamous cell carcinoma; **(C)** Resected left thumb distal phalangectomy specimen (H&E, ×4); **(D)** Resected left thumb specimen (H&E, ×10) demonstrating a malignant neoplasm with squamous differentiation, morphologically favoring trichilemmal carcinoma.

**Figure 3:**
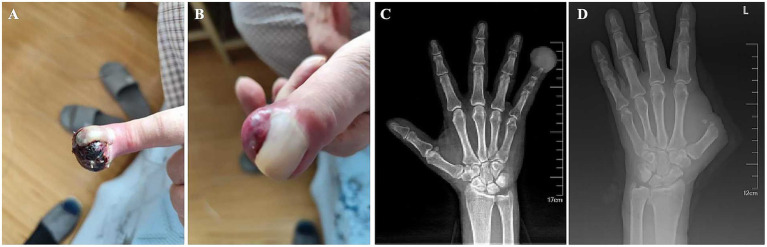
Clinical and radiological findings of the digital lesions. **(A)** Clinical photograph of right little finger showing erythematous swelling with distal necrosis; **(B)** Clinical photograph of the left thumb demonstrating nodular swelling; **(C)** Plain radiograph of the right little finger demonstrating osteolytic destruction of the distal phalanx; **(D)** Postoperative plain radiograph of the left thumb following distal phalangectomy.

**Figure 4:**
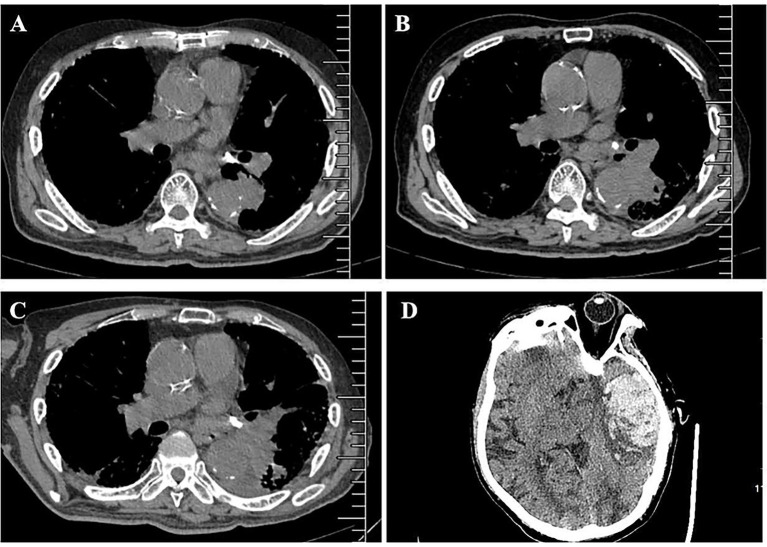
Serial chest CT images demonstrating rapid progression of a newly emerged soft-tissue mass in the left thoracotomy bed. **(A)** August 2025; **(B)** October 2025; **(C)** November 2025. **(D)** Non-contrast CT of the head (November 2025) demonstrating a large left fronto-parieto-temporal hemorrhage with significant midline shift, and scattered hyperdense nodular lesions within the brain parenchyma consistent with hemorrhagic cerebral metastases.

**Table 1:** Disease timeline.

Time	Event
August 2023	Diagnosis of left upper lobe SCC with concurrent IPF; VATS partial left upper lobectomy and mediastinal lymphadenectomy; pT2aN0M0 (Stage IIA)
August 2023–August 2024	Regular postoperative surveillance; tumor markers normal; CT: no recurrence or metastasis; no antifibrotic therapy initiated
September 2024	AE-IPF; PaO₂67 mmHg; KL-6 5,782 U/mL; methylprednisolone pulse HFNC; discharged on LTOT and nintedanib 150 mg bid
April–July 2025	Erythematous nodules on right little finger and left thumb; osteolytic destruction on plain radiographs; distal phalangectomy of left thumb; histology favoring trichilemmal carcinoma vs. acral metastasis
August 2025	Hepatic multiple metastases; abdominal wall subcutaneous nodule confirmed as metastatic SCC by biopsy; SCC-Ag 6.8 ng/mL
August–November 2025	Left thoracotomy bed recurrence with rapid progression on serial CT
November 2025	Cerebral hemorrhagic metastases; large left hemispheric hemorrhage (~60 mL); midline shift ~12 mm
December 2025	Death from transtentorial herniation

## Discussion

This case describes a rare and clinically instructive course in which a patient with early-stage lung SCC and co-existing IPF maintained stable disease for over one year following curative surgery, before developing explosive multi-organ metastasis within one year of an AE-IPF episode. The striking temporal association between AE-IPF-induced severe systemic hypoxia and the subsequent onset of multi-site metastasis provides important clinical evidence that the AE-IPF episode chronologically preceded the extensive metastatic spread, although whether AE-IPF contributed to or merely coincided with rapid tumor dissemination remains uncertain. The case also illuminates the extreme therapeutic challenges faced when treating lung cancer in the setting of IPF.

### AE-IPF-induced systemic hypoxia as a potential contributing factor to metastasis

The most compelling feature of this case is the temporal relationship between disease events: after more than one year of post-surgical stability, biopsy-confirmed multi-site metastasis emerged within approximately one year of AE-IPF onset, raising the possibility that AE-IPF may have represented a biological inflection point, although this remains speculative. The hallmark pathophysiological perturbation of AE-IPF is acute, severe hypoxemia, in this patient, PaO_2_ 67 mmHg with a PaO_2_/FiO_2_ ratio of approximately 160 mmHg persisting for several weeks representing not merely local pulmonary hypoxia, but profound systemic oxygen deprivation affecting all organs simultaneously.

Hypoxia-mediated tumor progression is a well-established phenomenon. Hypoxia drives tumor metastasis through multiple intersecting pathways: (1) upregulation of HIF-1α, which activates EMT transcriptional programs (Snail, ZEB1, and Twist, among others), causing tumor cells to lose epithelial polarity and acquire a migratory, invasive mesenchymal phenotype (7, 8); (2) HIF-1α-dependent induction of vascular endothelial growth factor (VEGF) and other angiogenic mediators, stimulating pathological neovascularization and providing a route for circulating tumor cells to reach distant organs (14); and (3) suppression of anti-tumor immune surveillance, including impaired cytotoxic activity of T-cells and natural killer (NK) cells through upregulation of inhibitory ligands and reduction of MHC class I expression, promoting immune evasion (7, 8). Notably, the contribution of high-dose corticosteroid therapy administered for AE-IPF to tumor progression cannot be excluded. Corticosteroids exert broad immunosuppressive effects, including inhibition of lymphocyte proliferation, impairment of NK cell function, and downregulation of pro-inflammatory cytokines, which may further blunt anti-tumor immune surveillance and accelerate metastatic spread. Critically, precancerous systemic organ priming by tumor-derived exosomes and soluble factors under hypoxic conditions can establish a “pre-metastatic niche”—an immunosuppressive, angiogenic, and ECM-remodeled microenvironment at distant sites that facilitates colonization and outgrowth of circulating tumor cells (15). While intratumoral hypoxia is a well-recognized driver of local invasion, AE-IPF uniquely delivers prolonged, severe, whole-body hypoxia—simultaneously exposing all potential metastatic target organs to pro-metastatic signals. We propose that this global hypoxic insult may have activated the aforementioned pro-metastatic cascades across multiple organs simultaneously, providing a mechanistic explanation for the near-synchronous appearance of liver, brain, abdominal wall, and digital metastases within a narrow time frame. Importantly, the systemic hypoxic insult did not occur in isolation. The high-dose systemic corticosteroids administered to treat AE-IPF exert broad immunosuppressive effects—including impairment of T-cell and natural killer–cell cytotoxic activity and attenuation of tumor immune surveillance—that may act synergistically with hypoxia-driven immune evasion to permit accelerated outgrowth of disseminated tumor cells. Hypoxia and corticosteroid exposure may thus represent convergent, mutually reinforcing contributors to the immunosuppressive milieu in this patient. Because these two factors coincided temporally and cannot be disentangled in a single case, corticosteroid-related immunosuppression must be regarded as a co-existing mechanism and an unavoidable confounder rather than excluded; the relative contribution of each remains undetermined. This observation carries direct clinical implications: for patients with lung cancer and concurrent IPF, aggressive prevention and management of AE-IPF to maintain adequate systemic oxygenation may be of critical importance.

### Rare metastatic patterns of lung squamous cell carcinoma

The abdominal wall subcutaneous metastasis in this case is a clinically significant and rare finding. The classical distant metastatic sites of lung SCC include the lung parenchyma (satellite or contralateral), bone, adrenal glands, and brain (16). Cutaneous and subcutaneous metastases from lung cancer are uncommon, occurring in approximately 1.8–11.8% of cases, and are predominantly located on the anterior chest wall; abdominal wall involvement is exceedingly rare (17, 18). A literature review identified only isolated case reports of abdominal wall metastases from lung SCC (17), underscoring the unusual nature of this presentation. The emergence of this atypical metastatic pattern may reflect AE-IPF-induced extreme phenotypic plasticity in tumor cells via EMT, endowing them with the capacity to colonize non-classical anatomical sites, combined with hypoxia-driven clonal selection of highly aggressive tumor subpopulations with enhanced invasive and ectopic homing capabilities (7, 8, 14).

Regarding the nature of the digital lesions, the histopathological report of the resected left thumb specimen favored a diagnosis of trichilemmal carcinoma (TC). TC is a rare malignant adnexal tumor of the outer root sheath of hair follicles that can exhibit histological overlap with well-differentiated SCC. Immunohistochemically, TC characteristically expresses CD34, CK17, and p53, which may assist in its distinction from metastatic SCC (19). However, several findings in this case cast significant doubt on the primary TC interpretation: (1) the patient had confirmed SCC metastases to the liver and abdominal wall appearing within the same time window; (2) osteolytic destruction of the distal phalanges is a radiographic feature strongly favoring metastatic disease over a primary cutaneous adnexal tumor; (3) acral metastasis to the hand and digits from lung SCC—though rare (incidence approximately 1%)—has been documented in the literature, with squamous cell carcinoma representing the most common histological subtype in phalangeal metastases (11, 12); and (4) SCC-Ag was elevated concurrently. Since the immunohistochemical panels applied in this case did not include specific markers for TC (CD34, CK17), a definitive pathological distinction between primary TC and metastatic SCC from the lung cannot be made. This diagnostic uncertainty is itself an important clinical lesson: any new cutaneous or subcutaneous nodule in a lung cancer patient—including lesions at uncommon sites such as the fingertips, should prompt thorough histopathological analysis with a comprehensive immunohistochemical panel to exclude metastatic disease. Critically, the discriminatory immunohistochemical markers required to resolve this distinction (CD34, CK17) could not be performed because of the limited residual biopsy tissue. We therefore regard the nature of this lesion as unresolved, and present it as a suspected acral metastasis that could not be definitively differentiated from a primary trichilemmal carcinoma. We explicitly acknowledge the potential for misclassification, and emphasize this diagnostic uncertainty as an instructive limitation rather than a confirmed metastatic event.

### The value of early antifibrotic therapy

Although this patient met clinical and radiological criteria for IPF at initial diagnosis in August 2023, antifibrotic therapy was not initiated until after AE-IPF occurred 13 months later. Both nintedanib and pirfenidone have been shown to slow the rate of FVC decline and reduce the risk of AE-IPF in patients with IPF (1, 9, 10). Nintedanib, a multikinase inhibitor targeting VEGF receptor, PDGF receptor, and FGF receptor signaling, additionally possesses antiangiogenic and potential anti-tumor properties (9). A case series has demonstrated that concurrent nintedanib and chemotherapy may be feasible and potentially protective against AE-ILD in patients with IPF and NSCLC (20). In this case, the absence of postoperative antifibrotic therapy may have allowed progressive fibrosis and increased vulnerability to AE-IPF. Earlier initiation of antifibrotic treatment might have reduced the risk of IPF progression or AE-IPF; however, this case cannot demonstrate that such therapy would have prevented metastatic dissemination. Furthermore, the high-dose corticosteroid therapy administered for AE-IPF should be considered as a potential confounding factor, given its possible contribution to tumor progression or impairment of antitumor immune surveillance. Taken together, these observations support the value of carefully considering antifibrotic therapy at the time of IPF diagnosis—including in perioperative or postoperative settings—while cautioning against overinterpreting its potential role in preventing tumor dissemination.

### Therapeutic challenges and the multidisciplinary approach

The treatment of lung cancer in patients with concurrent IPF represents one of the most challenging scenarios in thoracic oncology (3–5). All major therapeutic modalities carry heightened risks in this population:

- Surgery: While resection of early-stage disease is generally preferred, perioperative AE-IPF can occur in 4–23% of patients and carries a 30-day mortality exceeding 60% in some case series (3).
- Cytotoxic chemotherapy: Platinum-doublet regimens are associated with AE-ILD rates of 5–12%, with fatal outcomes reported in multiple prospective trials (4, 5). Carboplatin plus nab-paclitaxel appears to have a somewhat lower AE-ILD risk than other regimens.
- Immune checkpoint inhibitors (ICIs): Pre-existing ILD substantially increases the risk of ICI-related pneumonitis (incidence 19–43% in retrospective series compared to 6–10% in the non-ILD population), and the clinical differentiation of ICI pneumonitis from AE-ILD can be diagnostically challenging (13).
- Targeted therapy: Actionable oncogenic driver mutations (EGFR, ALK, ROS1, MET) are rarely detected in lung SCC, rendering targeted therapy largely inapplicable in this histological subtype (16).

By the time multi-site metastasis was detected in this patient, his ECOG (Eastern Cooperative Oncology Group) performance status had deteriorated to 4, making any systemic antitumor therapy intolerable. This trajectory illustrates the critical importance of proactive, preventive strategies rather than reactive management. We recommend that for patients with lung cancer and concurrent IPF, a dedicated MDT (Multi-Disciplinary Treatment) including pulmonologists, thoracic oncologists, thoracic surgeons, and palliative care specialists should be assembled at diagnosis and maintained throughout the disease course (3, 4). Postoperative surveillance should be more intensive than for lung cancer without IPF, incorporating not only tumor marker assessment and thoracic CT, but also systematic evaluation of IPF progression (HRCT, pulmonary function tests, serum KL-6), early warning signs of AE-IPF, and vigilant attention to any systemic symptoms that may predict metastatic disease at unusual sites.

### Limitations

This case report has the following inherent limitations. (1) As a single case, the temporal association between AE-IPF and explosive metastasis is hypothesis-generating and does not establish causation. (2) No patient-specific molecular evidence was obtained to support the proposed hypoxia-driven mechanism. The HIF-1α–driven EMT, angiogenic, and pre-metastatic-niche pathways invoked here are derived from the existing literature; confirmatory molecular and immunohistochemical profiling (e.g., HIF-1α, VEGF, EMT markers) of the patient’s tumor tissue could not be performed. The mechanistic model therefore remains speculative and requires validation in mechanistic and cohort studies. (3) The pathological work-up of the acral digital lesion was incomplete: discriminatory markers (CD34, CK17) were not performed because of limited residual material, precluding definitive differentiation between primary trichilemmal carcinoma and metastatic lung SCC. (4) Preoperative staging was incomplete—whole-body bone scan and PET-CT were not performed—which may have resulted in understaging and missed occult metastases. (5) The high-dose corticosteroids administered for AE-IPF carry intrinsic immunosuppressive effects that may have contributed to tumor progression, representing a significant confounder that cannot be disentangled from the hypoxia hypothesis in a single case.

## Conclusion

This case presents a rare and clinically instructive example of explosive multi-organ metastasis emerging shortly after AE-IPF in a patient with surgically resected early-stage lung SCC. The temporal association raises the hypothesis that AE-IPF-induced severe systemic hypoxia may contribute to tumor dissemination, conceivably through simultaneous activation of HIF-1α-driven EMT, pathological angiogenesis, immune evasion, and pre-metastatic niche formation across multiple organs. This case highlights several important clinical imperatives: early initiation of antifibrotic therapy at the time of IPF diagnosis to prevent AE-IPF; intensive, multidisciplinary postoperative surveillance strategies tailored to the unique risks of lung cancer with IPF; a high index of suspicion for metastatic disease at unusual anatomical sites; and the potential “dual-benefit” role of nintedanib in suppressing both fibrosis progression and tumor-promoting angiogenesis in this population. Future research should focus on prospective characterization of the relationship between AE-IPF, systemic hypoxia, and tumor metastasis kinetics in patients with lung cancer and IPF.

## Data Availability

The original contributions presented in the study are included in the article/supplementary material, further inquiries can be directed to the corresponding author/s.
